# Supplementary figures and images for: Multifunctional RNA-binding proteins influence mRNA abundance and translational efficiency of distinct sets of target genes
Source: PLoS Comput Biol. 2021 Dec 8;17(12):e1009658. doi: 10.1371/journal.pcbi.1009658 (PMC8687540; doi:10.1371/journal.pcbi.1009658)

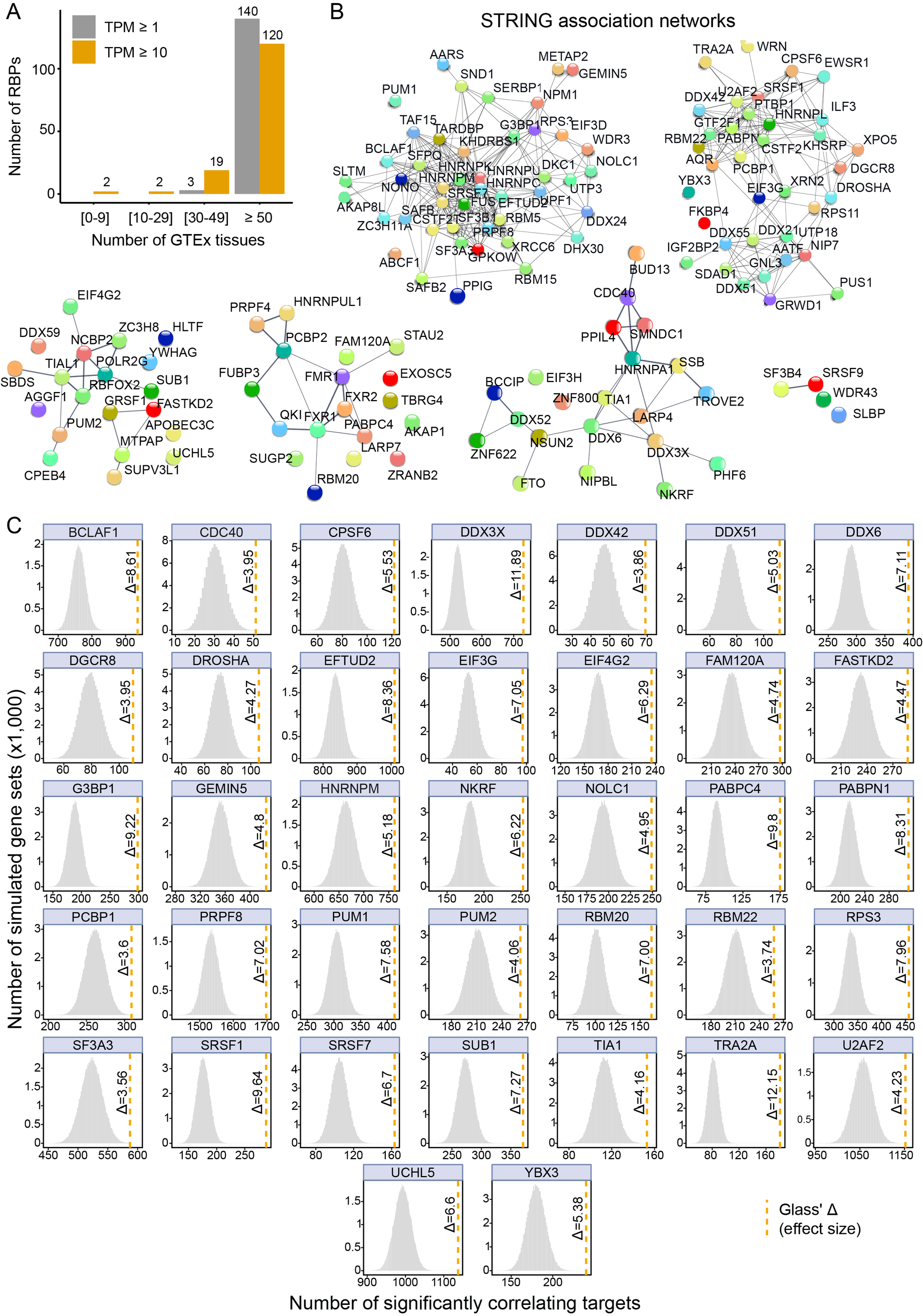

Supplement: S1 Fig — (A) Bar plot displaying the patterns of expression of the 143 RBPs across tissues. Average expression values in transcript per million (TPM) units were retrieved from the Genotype-Tissue Expression (GTEx) Project. Most of the RBPs are ubiquitously expressed across human tissues. (B) STRING protein-protein association networks from six coregulated RBP clusters (see also Fig 1A). Most of the clustered RBPs are involved in known functional interactions. (C) Heatmaps with Glass’ △ scores for all 37 TE-RBPs quantifying the effect size of the witness effects for significant TE correlations. (TIF) [file pcbi.1009658.s006.tif]

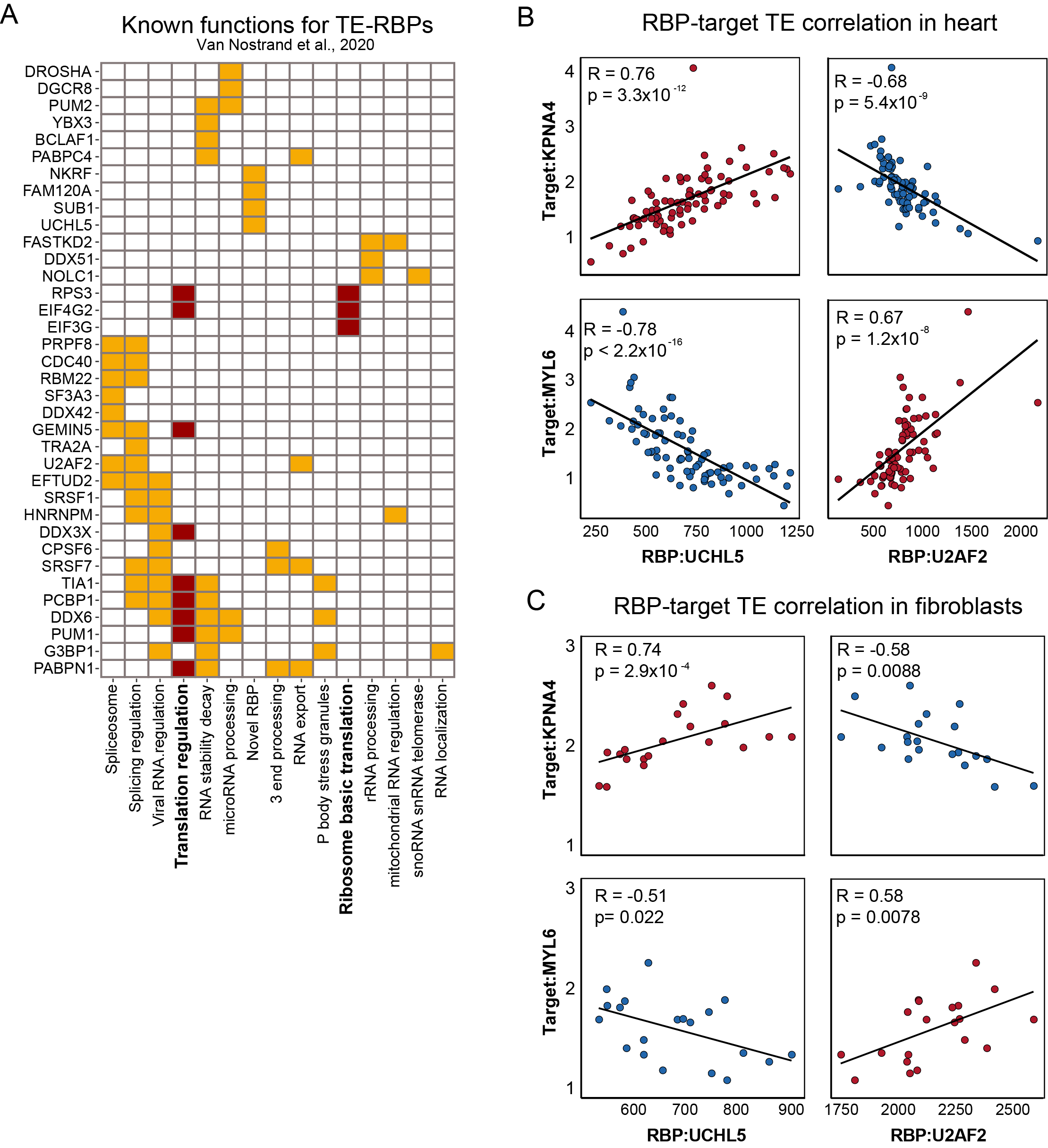

Supplement: S2 Fig — (A) Described functions by Van Nostrand et al. for the set of TE-RBPs. Functions related to translation (translation regulation and ribosome basic translation) are highlighted with dark red boxes. (B-C) Scatter plots representing the correlation of heart (B) and primary cardiac fibroblasts (C) translational efficiencies between UCHL5 and U2AF2 and two shared targets, KPNA4 and MYL6. UCHL5 and U2AF2 have marked opposite effects on their shared targets, indicative of a competitive effect replicated in two independent datasets. Scores and level of significance of the two Spearman’s correlations are displayed. (TIF) [file pcbi.1009658.s007.tif]

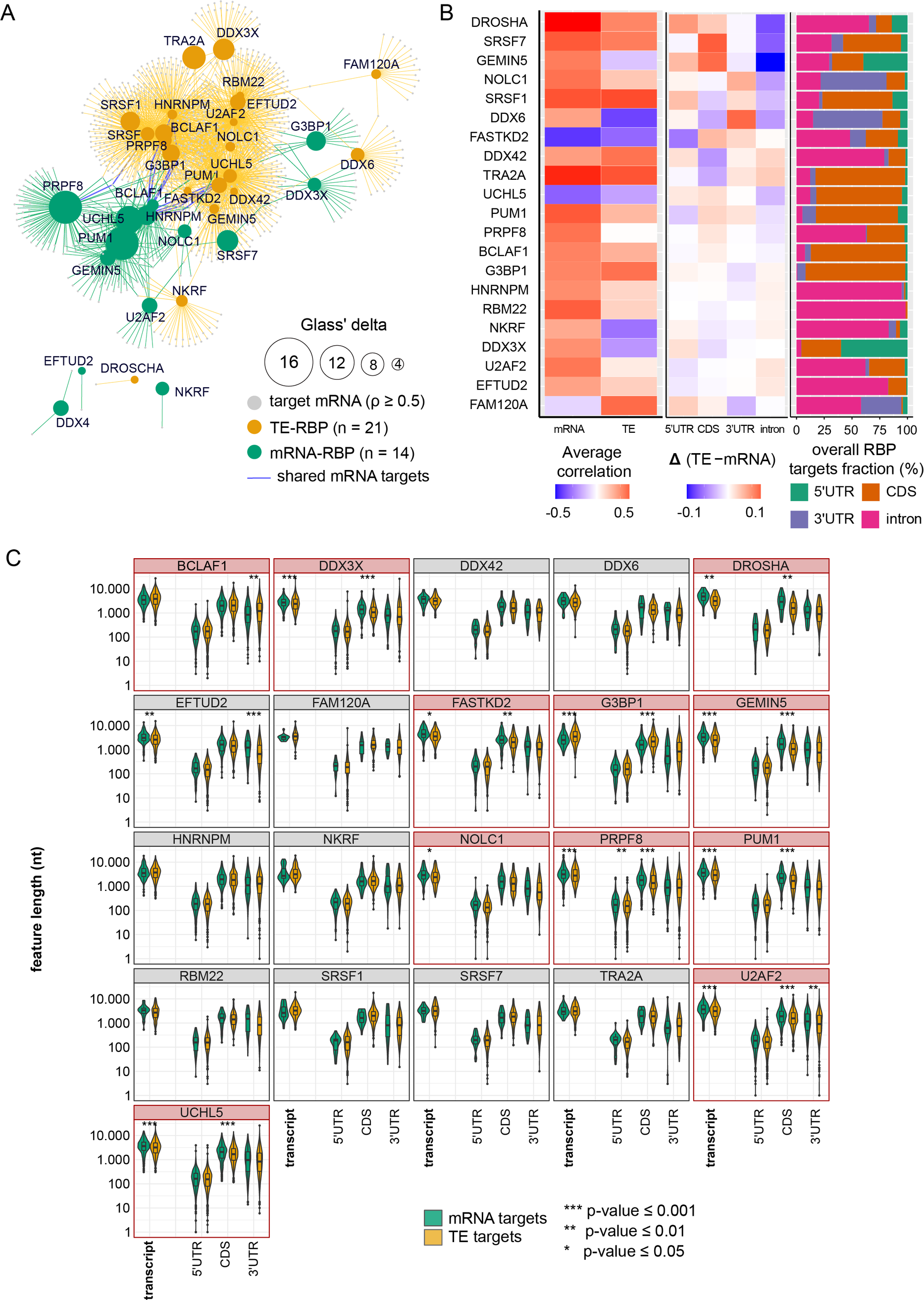

Supplement: S3 Fig — (A) Network representing multifunctional RBP-target interactions for both mRNA-RBPs (green) and TE-RBPs (brown) of strong correlating pairs. Blue lines indicate shared targets in both mRNA abundance and TE regulation of the same RBP. (B) Left: heatmap representing the average mRNA and TE RBP-target correlation values for all 21 multifunctional RBPs. Middle: heatmap representing differences in the relative proportion of feature binding sites (TE-mRNA) for all 21 multifunctional RBPs. Right: bar plot showing the overall proportion of feature binding sites for all 21 multifunctional RBPs. (C) Box plots with 5’ UTR, CDS, and 3’ UTR sequence lengths in nucleotides for mRNA and TE targets corresponding to the set of 21 multifunctional RBPs. For each target gene, the most abundant isoform is represented. (TIF) [file pcbi.1009658.s008.tif]

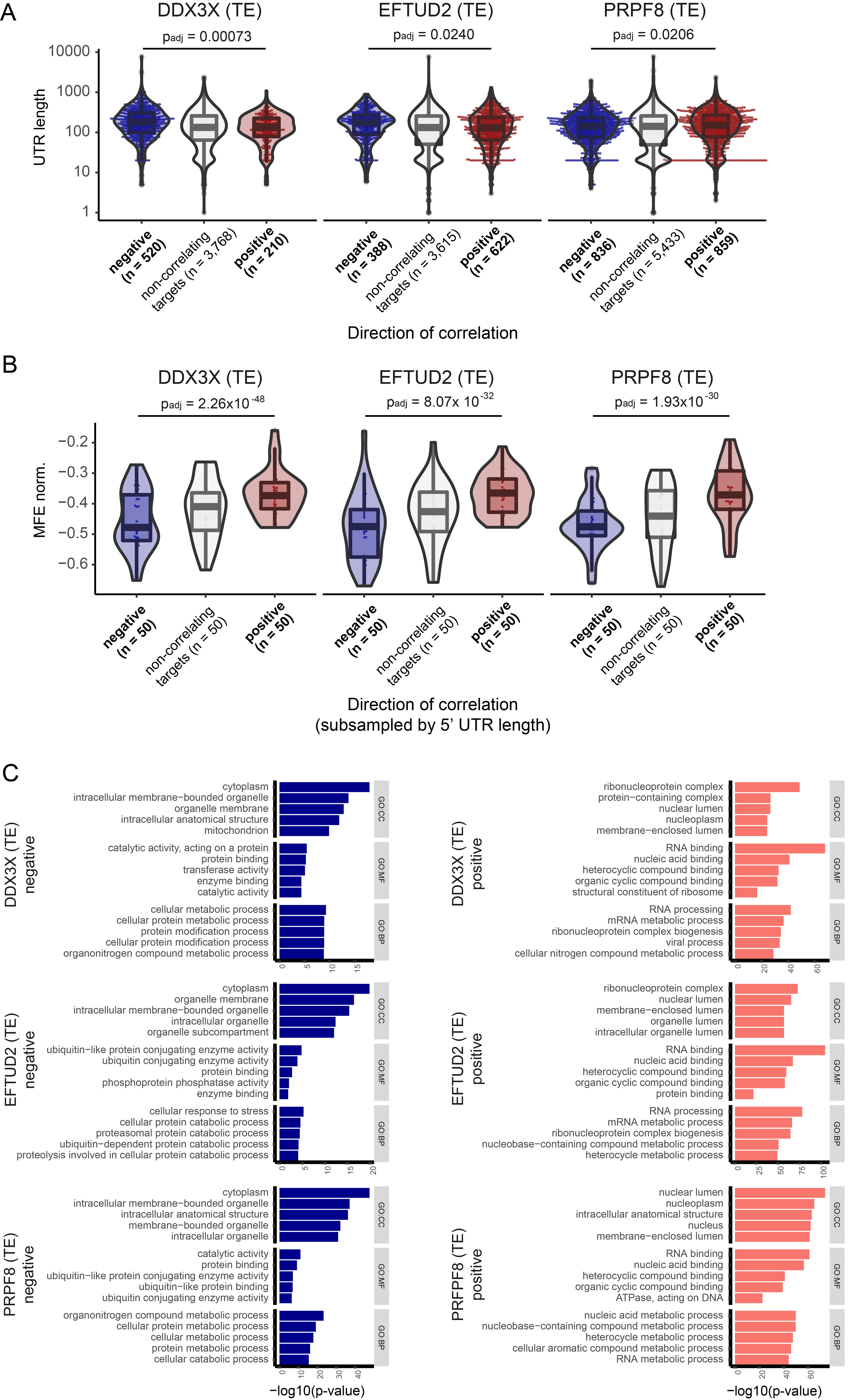

Supplement: S4 Fig — (A) Box and violin plots with 5’ UTR lengths for positively and negatively correlated TE targets corresponding to DDX3X, EFTUD2, and PRPF8. (B) Box and violin plots with length normalized MFE scores for positively and negatively correlated TE targets. We subsampled sets of 50 genes per group and RBP, so each of the groups had a similar distribution of 5’ UTR lengths. For comparison, non-correlating target genes were included in the panel figure. (C) Enriched GO terms in the sets of positive and negative correlating targets for DDX3X, EFTUD2, and PRPF8. For each RBP, the 5 most significant GO terms are displayed. (TIF) [file pcbi.1009658.s009.tif]
