# Supplementary material for: Multifunctional RNA-binding proteins influence mRNA abundance and translational efficiency of distinct sets of target genes
Source: PLoS Comput Biol. 2021 Dec 8;17(12):e1009658. doi: 10.1371/journal.pcbi.1009658 (PMC8687540; doi:10.1371/journal.pcbi.1009658)
Supplement: Attachment — Submitted filename: Review.docx [file pcbi.1009658.s010.docx]

**Review of manuscript: “Multifunctional RNA-binding proteins influence mRNA abundance and translational efficiency of distinct sets of target genes”**

In this manuscript, the authors study the role of 143 RNA-binding proteins (RBPs) expressed in the heart, in regulating the mRNA abundance and translation efficiency of their CLIP-derived target genes using previously published [1] matched RNA-Seq and Ribo-Seq data from 80 human hearts. The authors were able to identify significant associations of RBP abundance with target genes, with 58 RBPs (referred to as mRNA-RBPs) associated with mRNA abundance and 37 RBPs (referred to as TE-RBPs) with translational efficiency of target genes. Of the 37 TE-RBPs, they were able to confirm similar associations for 27 of them in an independent set of translatomes of primary cardiac fibroblasts [2], suggesting their role in cardiac translation regulation.

Further, they identified a subset of 21 RBPs which were associated with both mRNA abundance and translational efficiency but interestingly with limited overlap of target genes of the associated with the two traits. They call these ‘multi-functional’ RBPs where the role of the RBPs is dependant on the mRNA target. These distinct sets of target genes were found to be enriched for separate biological processes. Additionally, binding affinity and complexity for mRNA structures like 5’ UTR, CDS, etc were evaluated and a subset of multifunctional RBPs like DDX3X, UCHL1 were found to have differential binding affinity to target length and/or 5’ UTR length suggesting that this could be how the RBPs regulate the two target sets differently.

The study is well-structured and logical with a sound methods and

discussion addressing limitations and questions raised by their results. The study highlights the complexity of gene expression regulation where RBP regulation is not limited to regulating mRNA abundance, in turn affecting translational efficiency, but with distinct target genes being regulated with independent biological outcomes and is of interest for readers of PLOS Computational Biology.

However, this referee has the following suggestions for the authors to address.

1. The study has identified target genes for regulation for mRNA abundance or translational efficiency by RBPs and potential mechanisms of such regulation too. The study would really benefit from experimental validation of some of these results. A quick search on GEO yielded RNA-Seq datasets of RBM20 deletion in iPS-derived cardiomyocytes (GSE175886). It would be of interest to see if the RBM20 deletion affects the target genes as predicted. Is the dominant isoform of *TTN* different in these cardiomyocytes? There is another recently published paper [3] which looks at translational efficiency of DDX3X and contains RNA-Seq datasets of DDX3X loss-of-function mutation in lymphoma cell lines. Although not the best fit for validation, some of the target genes are expected to be conserved across tissues too. If the authors can show that deletion of the RBP expression would dysregulate the translatome as suggested from this study’s results, it would strengthen the paper greatly. This referee recommends that the above two datasets and/or a few other datasets of relevance be analysed for independent validation.
2. Make the manuscript easier to digest for the reader by providing brief explanations of key concepts or measures used throughout the paper at first mention, for example for mRNA abundance versus translational efficiency, Glass’ Delta, generation of theoretical distribution to compare against, minimum free energy.

1.	van Heesch S, Witte F, Schneider-Lunitz V, Schulz JF, Adami E, Faber AB, et al. The Translational Landscape of the Human Heart. Cell. 2019;178: 242-260.e29. doi:10.1016/j.cell.2019.05.010

2.	Chothani S, Schäfer S, Adami E, Viswanathan S, Widjaja AA, Langley SR, et al. Widespread Translational Control of Fibrosis in the Human Heart by RNA-Binding Proteins. Circulation. 2019;140: 937–951. doi:10.1161/CIRCULATIONAHA.119.039596

3.	Gong C, Krupka JA, Gao J, Grigoropoulos NF, Giotopoulos G, Asby R, et al. Sequential inverse dysregulation of the RNA helicases DDX3X and DDX3Y facilitates MYC-driven lymphomagenesis. Molecular Cell. 2021 [cited 7 Sep 2021]. doi:10.1016/j.molcel.2021.07.041
