# Supplementary material for: Multifunctional RNA-binding proteins influence mRNA abundance and translational efficiency of distinct sets of target genes
Source: PLoS Comput Biol. 2021 Dec 8;17(12):e1009658. doi: 10.1371/journal.pcbi.1009658 (PMC8687540; doi:10.1371/journal.pcbi.1009658)
Supplement: Attachment — Submitted filename: Response_to_reviewers.docx [file pcbi.1009658.s011.docx]

Dear Dr. Tucker-Kellogg and Dr. Beard,

Thank you for sending us the overall highly positive feedback of the reviewers and for welcoming a revised submission of our manuscript. We have now addressed the comments of the reviewers below and included the suggested changes in the revised manuscript. All figure files have been checked for meeting the PLOS requirements and all data and scripts used for the study are publicly available. We hope for a swift publication in PLOS Computational Biology.

With kind regards,

(On behalf of all authors)

Sebastiaan van Heesch

**--Response to reviewers--**

PCOMPBIOL-D-21-01425

**Multifunctional RNA-binding proteins influence mRNA abundance and translational efficiency of distinct sets of target genes**

***Reviewer comments in black, author response in blue writing.***

**Reviewer #1**

In this manuscript, the authors study the role of 143 RNA-binding proteins (RBPs) expressed in the heart, in regulating the mRNA abundance and translation efficiency of their CLIP-derived target genes using previously published [1] matched RNA-Seq and Ribo-Seq data from 80 human hearts. The authors were able to identify significant associations of RBP abundance with target genes, with 58 RBPs (referred to as mRNA-RBPs) associated with mRNA abundance and 37 RBPs (referred to as TE-RBPs) with translational efficiency of target genes. Of the 37 TE-RBPs, they were able to confirm similar associations for 27 of them in an independent set of translatomes of primary cardiac fibroblasts [2], suggesting their role in cardiac translation regulation.

Further, they identified a subset of 21 RBPs which were associated with both mRNA abundance and translational efficiency but interestingly with limited overlap of target genes of the associated with the two traits. They call these ‘multi-functional’ RBPs where the role of the RBPs is dependent on the mRNA target. These distinct sets of target genes were found to be enriched for separate biological processes. Additionally, binding affinity and complexity for mRNA structures like 5’ UTR, CDS, etc were evaluated and a subset of multifunctional RBPs like DDX3X, UCHL1 were found to have differential binding affinity to target length and/or 5’ UTR length suggesting that this could be how the RBPs regulate the two target sets differently.

The study is well-structured and logical with a sound methods and discussion addressing limitations and questions raised by their results. The study highlights the complexity of gene expression regulation where RBP regulation is not limited to regulating mRNA abundance, in turn affecting translational efficiency, but with distinct target genes being regulated with independent biological outcomes and is of interest for readers of PLOS Computational Biology.

However, this referee has the following suggestions for the authors to address.

The study has identified target genes for regulation for mRNA abundance or translational efficiency by RBPs and potential mechanisms of such regulation too. The study would really benefit from experimental validation of some of these results. A quick search on GEO yielded RNA-Seq datasets of RBM20 deletion in iPS-derived cardiomyocytes (GSE175886). It would be of interest to see if the RBM20 deletion affects the target genes as predicted. Is the dominant isoform of TTN different in these cardiomyocytes? There is another recently published paper [3] which looks at translational efficiency of DDX3X and contains RNA-Seq datasets of DDX3X loss-of-function mutation in lymphoma cell lines. Although not the best fit for validation, some of the target genes are expected to be conserved across tissues too. If the authors can show that deletion of the RBP expression would dysregulate the translatome as suggested from this study’s results, it would strengthen the paper greatly. This referee recommends that the above two datasets and/or a few other datasets of relevance be analysed for independent validation.

We thank the reviewer for suggesting these two datasets for validation. The RBM20 deletion datasets - including GSE175886 and sequencing data of previous rat and mouse KO models generated by our lab - used RNA-seq and did not perform matched ribosome profiling experiments to evaluate how RBM20 may impact translational efficiency. Our lab has previously demonstrated how RBM20 represses splicing, and thereby orchestrates isoform abundance of the same set of sarcomere genes we highlight in our current manuscript (Guo et al., *Nat. Med.* 2012; Maatz et al., *JCI* 2014). The effects of RBM20 modulation on isoform usage, and how this contributes to transcriptional isoform switches that drive dilated cardiomyopathy in rodents, dogs, and humans, are well understood and fully in line with our results. We have clarified this in the revised version of the manuscript (Page 10). Our current study is the first to demonstrate that RBM20 expression also directly correlates with the protein synthesis rates of TTN and many other RBM20-targeted sarcomere genes. We did recently report that *TTN* isoform-specific changes in translational efficiency exist (Figure S3K-N of van Heesch, *Cell* 2019) but had previously not been able to statistically connect this to regulation by RBM20, as we now successfully report here.

The reviewer suggested a second dataset where DDX3X knockdowns were evaluated in lymphoma cell lines. In this study from Gong et al. (PMID: 34437837), the authors found 70 and 90 genes whose translational efficiencies were up- or down-regulated, respectively, after knockdown of DDX3X. However, they did not find enrichment of DDX3X eCLIP targets among the 70 upregulated genes, and consequently we would not expect to find a high overlap with our study. From the set of 90 translationally downregulated genes at the level of translation, the authors found an enrichment of eCLIP targets associated with functions in translation, and more specifically, ribonucleoproteins (RNPs). Such findings were validated by the authors using proteomic profiling. To corroborate these observations, in our updated manuscript we similarly searched for enriched GO terms for DDX3X targets (as well as EFTUD2 and PRPF8 targets) separated by directionality of regulation (up- or downregulated). We indeed also find that targets positively correlating with DDX3X are enriched in RNPs, being the most significant cellular component GO term. We have added these lists of GO terms in a new Figure (Figure S4C; see Figure A below for DDX3X) and discuss how our results are in line with the main findings from Gong et al. (Page 9, last paragraph of Results).

**Figure A.** Enriched GO terms in the sets of positive and negative correlating targets for DDX3X.

Make the manuscript easier to digest for the reader by providing brief explanations of key concepts or measures used throughout the paper at first mention, for example for mRNA abundance versus translational efficiency, Glass’ Delta, generation of theoretical distribution to compare against, minimum free energy.

We have revised the text carefully and included brief explanations for the concepts introduced in the paper along the Results section. Definitions of ‘mRNA abundance’ and ‘translational efficiency’ were included in Page 3. We expanded the sentence that described the generation of the theoretical distribution in the Results (Page 4, first paragraph). Brief explanations of the ‘Glass’ delta score’ and ‘calculated percent spliced in (PSI)’ concepts were included in Page 4. Finally, the ‘minimum free energy (MFE)’ measure was introduced in Page 9.

**Reviewer #2:** 
Reviewer #2: Multifunctional RNA-binding proteins influence mRNA abundance and translational efficiency of distinct sets of target genes
by Schneider-Lunitz et al.

Schneider-Lunitz et al. combine the transcriptomes and translatomes of human hearts with the target preferences of human RBPs to deduce potential associations and identify new multi-functional regulators of mRNA stability and translation. Genome-wide measurements for mRNA abundance and translational efficiency (derived from matched Ribo-seq and RNA-seq) for 80 human hearts were taken from the authors’ previous study (Van Heesch et al, Cell, 2019). RBP targets were extracted from publicly available eCLIP data for 142 RBPs, plus RBM20 from an independent dataset. Using correlation analysis (Spearman), the authors identify significant associations between the expression of a given RBP and the mRNA abundance (58 RBP-mRNA associations) or translation efficiency (37 RBP-TE associations) of its targets. They find that many RBPs show shared modes of regulation, having overlapping targets with opposing or concordant directionality of regulation. Moreover, they identify 21 RBPs as multifunctional RBPs, which influence mRNA abundance and translational efficiency for different subsets of target RNAs.

Overall, this is an interesting and very well performed study. The manuscript is well written and the figures are of high quality. The analyses are based on solid data and statistical approaches. Specific points that would enhance the impact of the study are listed below.

**Major comment:**

Based on the high similarity in the regulatory patterns of some RBPs (Figure 2AB), the question arises whether such results could be influenced by colinearity in the RBPs’ expression and/or a certain fuzziness in the assignment of the target genes for some RBPs. The impact of colinearity could for instance be assessed by partial correlations for pairs of similar RBPs to test whether RBP1 still shows a significant association with its target genes when correlations are corrected for RBP2. Regarding the second factor, the validity of target gene assignment for a given RBP could be assessed by cross-testing target genes, i.e., correlating the target genes of RBP1 against RBP2 and vice versa.

We thank the reviewer for pointing out the possible effects of collinearity and fuzziness in the set of RBPs with high similarity of correlation with their target genes. RBPs form part of complex regulatory networks and also bind and modulate each other's transcription and translation levels, resulting in a highly complex system where it becomes challenging to define hierarchy.

The selected parameters to define the initial set of RBP targets were based on experimental CLIP data and defined using the cut-offs proposed by the labs that generated the data, to minimize the number of false positive target identifications. Thus, the significance of the observed correlations is restricted to the subset of genes that we initially defined as RBP targets, based on these CLIP data. By generating 100,000 matched sets of simulated target genes, we further ensured that the observed correlations between the investigated RBPs and CLIP-derived targets were significant and could not be explained by chance. However, this sampling analysis *did* reveal that individual associations in the simulated sets can yield significant correlations by chance (NB.: these distributions are plotted as histograms in e.g., Figure 1B and S1C). Together with the Glass delta, this gave us a reliable estimation of the approximate FDR of target assignment for each of the investigated RBP-target set associations (Figure S1C). We realize that a certain level of fuzziness in target assignment is thus inevitable, especially for RBPs that have general roles and consequently bind many target genes. This becomes particularly important when our results will be interpreted by others to investigate individual RBP-target connections, which will require additional experimental validations. We have therefore more clearly addressed this in the discussion of the revised manuscript.

Reassuringly, the specificity of our approach is strengthened by our observations for the muscle specific RBP RBM20, for which all known targeted sarcomere genes are among the top correlating hits (Figure 2C). These hits have been extensively validated by our lab in previous studies across multiple species and conditions (Guo et al., Nature Med 2012; Maatz et al., JCI 2014). Similarly, multiple independent investigations into the modes of action of DDX3X in different systems (Calviello et al., NAR 2021 and Gong et al., Mol Cell 2021) point toward regulation of the same sets of target genes in a similar mechanistic fashion as observed in our results.

Following the Reviewer’s suggestion, we have additionally evaluated the impact of collinearity by assessing partial correlations of RBP pairs on shared target genes. We initially selected the 6 clusters of highly correlated RBPs identified in Figure 2B and calculated the fraction of target genes that are still significant after correcting for partial correlations, considering all possible RBP-RBP pairwise comparisons inside each cluster. For 3 of these clusters, all the target genes were still significant after correction for RBP collinearity, while only 6-16% of the target genes were no longer significantly associated with the RBPs after this correction for the remaining 3 clusters. This indicates that collinearity is unlikely to drive the observed correlations in RBPs with high numbers of shared target genes. We have added this analysis and findings to Figure 2B (see Figure B below) and to the Methods section.

**Figure B.** Dendrogram with hierarchically clustered TE-RBPs based on pairwise RBP-RBP overlaps. Shared target genes of all paired RBPs were included for clustering.  Bottom heatmaps with translational efficiency correlations of selected RBP clusters and shared significant targets. Pie charts illustrate the fraction of targets that remain significant after correcting for cluster RBP collinearity.

**Minor comments:**

Figure 2A: How many and which genes went into this analysis? Is this the union of target genes of all RBPs or some selection?
We included any known RBP target gene whose translational efficiency (TE) showed a significant correlation with the expression dynamics of one or more TE-RBPs (n = 37), which were previously shown to bind these targets based on CLIP experiments. For all 37 TE-RBPs combined, this results in the inclusion of 6,153 unique target genes. We have clarified this in the revised version of the manuscript: in Figure 2A itself, and in the caption of Figure 2.

“For instance, depending on the shared target gene bound by the splicing factor U2AF2 and the protease UCHL5, completely opposite effects on TE could be observed and independently replicated (Figure 2C, Figure S2B and S2C).”
Reference to Figure 2C seems wrong.
The reviewer is correct, this sentence should have referenced Figure 2B instead of 2C. We have corrected this in the revised manuscript.

Figure 2D/E: Extend legends to clarify what units were used for the scatter plots.
We have added the unit description for the scatter plots in the revised manuscript figure 2D/E itself and the figure caption. The scatter plots in Figure 2D display the correlation between RBM20 expression as measured by Ribo-seq (normalized counts; x-axis) and the translational efficiency of two sarcomere genes, TNNI3K and TTN (y-axis). The scatter plot in Figure 2E shows the correlation between RBM20 expression levels as measured by Ribo-seq (normalized counts) and the percentage spliced-in (PSI) of TTN exon:156.

Figure S3B: This figure requires more explanation. What exactly is shown in the middle panel? It seems that some RBPs show a region-dependent effect. For instance, as expected, SRSF7 seems to tend to regulate mRNA abundance via intronic binding and translational efficiency via CDS binding.

The reviewer is correct, and we have added a more detailed explanation of Figure S3B in the Results section (Page 9, first paragraph). In summary, Figure S3B is subdivided into three panels that display the following:

The **left** panel displays a heatmap that shows the average correlation between RBP expression and target gene mRNA abundance and TE, for all 21 multifunctional RBPs. Examples like FASTKD2 and UCHL5 appear (on average) to be negative regulators of their targets (for both quantitative traits), while SRSF7 binding appears to nearly exclusively have positive effects.

The **middle** panel displays a heatmap that visualizes the relative proportion of feature binding sites per transcript features (5’ UTR, CDS, 3’ UTR and intron), with a focus on the difference in binding location between targets regulated on the transcriptional or translational level. The goal of this heatmap was to investigate whether the position of binding is related to the effect of the RBP. For instance, 5’ UTR binding could have stronger implications for translation, whereas an enrichment for introns could indicate a role in transcriptional processes (e.g., pre-mRNA splicing). As correctly pointed out by the reviewer, for RBPs like SRSF7 or GEMIN5, the results indicate that for targets whose TE is influenced by SRSF7 or GEMIN5 binding, the RBP-mRNA interaction is more likely to take place within exonic CDS regions (red indicates higher in TE targets than in mRNA targets). For targets of these same RBPs whose transcript levels are influenced, binding appears enriched in intronic regions (blue indicates higher in mRNA targets than in TE targets). For DDX6, a similarly strong difference seems to indicate that effects of DDX6 binding on translational efficiency are mediated through 3’ UTR binding.

The **right** panel contains a bar plot that shows the overall proportion of feature binding sites for all 21 multifunctional RBPs. SRSF7 largely binds to intronic and CDS regions while DDX6 binds to 3’ UTR regions.

Although we realize this figure panel is convoluted and complex, we deemed it necessary to provide visually interpretable information on combinations, ratios, and consequences of binding preferences and binding effects across the entire set of studied RBP-mRNA interactions.

Figure 4B: MFE values have been normalised by length, but it is unclear whether this really works. The authors should check for differences in lengths between categories and use length-matched subsamples if needed. Also, what is the MFE distribution for non-correlated genes for comparison?

We have included a new supplementary figure (Figure S4) to illustrate the distribution of 5’ UTR lengths between categories for the three selected RBPs. We found minor significant differences in UTR length (see Figure C). These differences warrant our normalization efforts, since MFE is affected by sequence length. The difference in MFE normalized by length is much greater than the small difference observed in UTR length, so it becomes clear that UTR length is not the primary determinant of the observed effect. As suggested by the Reviewer, we generated length-matched subsamples for every RBP, including 50 genes for each group (negative correlation, non-correlating, positive correlation) and we observed similar significant differences between positively and negatively correlating targets (see Figure C, bottom), confirming that UTR length does not drive the observed differences. For comparison, we have also included the distribution of normalized MFE values and 5’ UTR lengths for non-correlating target genes in Figures 4B and S4. Non-correlating targets displayed intermediate MFE and UTR lengths compared to positively and negatively correlating targets.

**Figure C. (Top)** Box and violin plots with length normalized MFE scores for positively and negatively correlated TE targets corresponding to the three selected multifunctional RBPs with the highest significance in **Figure 4A** (DDX3X, EFTUD2, PRPF8). **(Middle)** Box and violin plots with 5’ UTR lengths for positively and negatively correlated TE targets corresponding to DDX3X, EFTUD2, and PRPF8. **(Bottom)** Box and violin plots with length normalized MFE scores for positively and negatively correlated TE targets. We subsampled sets of 50 genes per group and RBP, so each of the groups had the same 5’ UTR length. For comparison, non-correlating target genes were included in the panel figure.

**Reviewer #3:**

This manuscript presents a bioinformatics study investigating 143 RNA-binding proteins’ (RBPs) transcriptional and translational regulation roles in human heart. The authors re-analyzed previously published paired Ribo-seq and RNA-seq data from 80 human hearts and interrogated the correlation between expression levels of the 143 cardiac-expressed RBPs and the expression level and translational efficiency (TE) of their corresponding RNA-binding targets (obtained from ENCODE database, CLIP-seq approaches). In total, 58 RBPs were found to have significant associations with target mRNA abundance (denoted as “mRNA-RBPs”) and 37 RBPs with significant associations with target translational efficiencies (denoted as “TE-RBPs”), among which 21 RBPs were found in both categories and denoted as multifunctional RBPs. These includes 27 novel TE-RBPs, previously unknown for translational control process, including a muscle-specific and dilated cardiomyopathy-associated splicing regulator RBM20. The authors showed the multifunctional RBPs are context-specific where their functional outcome depends on the set of mRNAs they target, and their target genes also appear to be regulated independently, facilitated by a differential affinity for coding sequence length or 5’ UTR structure. In summary, this study gives a comprehensive analysis on the RBP regulatory roles in human heart based on current available transcriptome, translational efficiency and RNA-binding data with rigorous experiment design and statistical tests. It provides a list of potential TE-RBPs, multifunctional RBPs and many interesting insights of how these RBPs regulating the expression and translation, which could be further validated and studied in this field.

**Major Comment:**
This study comes up with a very interesting sets of RBPs that potentially participate in the expression and translation control of the mRNAs and provides the directionality information as well. I was wondering how this list overlaps with previously published screen studies that focused on the RBP functions and that also showed directionality, such as the study Luo et al. in Nature Structural & Molecular Biology (2020) and other similar studies.

We thank the reviewer for the suggestion to compare our findings with previously published studies. We included two RBP-focused studies describing screens that would fit for this purpose: the Luo et al. study (as proposed by the reviewer) and a study by Chothani et al., which investigates the roles of RBPs in fibrosis (PMID: 31284728).

The study from Luo et al. screened 690 RBPs using a luciferase-based tethered function assay (TFA), which recruits RBPs to the 3’ UTR and measures the effect of RBP binding on mRNA stability and/or translation using two different reporter constructs. The authors identified a total of 320 significant RBPs using either the Renilla (n = 299) or *firefly* (n = 71) reporter systems, indicating that only 50 RBPs were determined to be significant in both reporter contexts. From our set of 74 RBPs with significant associations with target mRNA abundance (mRNA-RBPs) and/or translational efficiencies (TE-RBPs), 35 mRNA-RBPs and 19 TE-RBPs were present in the list of 690 RBPs initially tested by Luo et al. Of these, 10 mRNA-RBPs (STAU2, FTO, XRCC6, EIF3D, LARP4, GTF2F1, SAFB2, DHX30, TARDBP, ZRANB2), 2 TE-RBPs (DGCR8, CPSF6), and 5 multifunctional RBPs (G3BP1, UCHL5, TRA2A, U2AF2, and RBM22) were found to be significant in one of the two reporter assays. We have added this information to supplemental table 1.

A single multifunctional RBP (DDX6) identified by our study was found to be significant in both renilla and firefly reporter assays. DDX6 also displayed identical directionality between both studies: It showed a prominent negative effect in both TFAs, and we similarly found that the translational efficiencies of 87% of DDX6 targets correlated negatively. Moreover, DDX6 was preferentially recruited to 3’ UTRs of correlating target genes (3’ UTR binding at > 63% of its targets). This enrichment for 3’ UTRs was also observed by Luo et al., who assign the repressive effects of DDX6 through 3’ UTR binding to its suggested role in the closed-loop formation of translated mRNAs. We have updated the revised manuscript describing the observed reproducibility between both studies with regard to the role of DDX6 in regulating translation (Page 9, first paragraph).

The endogenous preference of an RBP to bind a target in a specific region of the RNA molecule appears to be a critical factor for the observed reproducibility between both studies: most RBPs that we find to influence translation **do not** bind 3’ UTRs (Figure S4B). Although this is clearly acknowledged as a limitation of 3’ UTR TFAs (Luo et al., Supplementary Note, 2nd paragraph), it suggests that future reporter assays for assessing the roles of RBP in translation would best be complemented by *in vivo* read outs of translation (as provided by our current study) and recruitment strategies to different sites of mRNAs (e.g., 5’ UTRs or CDS).

The similarity in observations between our study and the study published by Chothani et al. is currently described in Figure 1C. In this study, the authors defined the frequency with which RBP abundance correlated with target gene translational efficiency in fibrosis, through generation and analysis of a cohort of 20 primary cardiac fibroblast translatomes. Despite differences in source material (primary fibroblasts vs. heart tissue) and sample numbers used to calculate the correlations (20 vs. 80 samples), for 25 out of 37 TE-RBPs we could replicate the significance of the RBP associations with target translational efficiencies (Figure 1C).

**Minor comments:**
In the “Multifunctional RBPs including DDX3X and G3BP1 regulate mRNA abundance and translational efficiency of independent sets of target genes” of Results section, I have some problem in understanding the expression “which decreased further for the most strongly correlating targets” (line 11 of the paragraph) and Figure S3 A. I was wondering if the author could explain a bit more, highlight the “most strongly correlating targets” in the corresponding figure or something that can help readers better understand the results.

We agree with the reviewer that this statement was confusing and have removed it from the manuscript. Figure S3A referred to a figure panel that previously showed that an increase of the stringency of the correlation threshold (by applying a consistent r > 0.5 cut-off, in addition to the significance cut-off that was already applied), further decreased the overlap between the sets of most strongly correlating targets. In other words, the more significant the correlation between the RBP and regulation of a specific target (mRNA abundance *or* TE), the less likely it was that a single target gene was regulated by the same RBP on both levels of gene expression control (mRNA abundance *and* TE). As these results were highly consistent with the results currently displayed in Figure 3A, this panel was no longer part of the submitted manuscript and we apologize for this inconvenience.

In the discussion, the authors mentioned all discovered multifunctional RBPs are ubiquitously expressed across human tissues, but I did not find the data or plots presenting this result in the manuscript. I suggest it would be great if the authors could show the results in the main figures or supplementary figures (e.g. using heatmap and GTEx data).

The expression of RBPs across the GTEx tissues is given in Figure S1A, where we visualized a bar plot that displays the expression of the 143 RBPs across all GTEx tissues. This shows that 140 out of 143 RBPs are expressed with a TPM ≥1, and 120 out of 143 show an expression of TPM ≥ 10 in at least 50 GTEx tissues. Nineteen other RBPs, summing up to a total of 139 out of 143 RBPs, have a TPM ≥ 10 in at least 30 GTEx tissues. The 4 RBPs that showed more tissue-restricted expression patterns (RBM20, ZC3H11A, RBM15 and ZC3H8) were all expressed in the human heart. In the revised manuscript, we have added an additional reference to Figure S1A in the discussion and the methods section that describes the use of the GTEx data.
